# The Impact of Incentives on Job Performance, Business Cycle, and Population Health in Emerging Economies

**DOI:** 10.3389/fpubh.2021.778101

**Published:** 2022-02-10

**Authors:** Wei Liu, Yaoping Liu

**Affiliations:** ^1^Department of Business Administration, International College, Rajamangala University of Technology Krungthep, Bangkok, Thailand; ^2^Department of Global Buddhism, Institute of Science Innovation and Culture, Rajamangala University of Technology Krungthep, Bangkok, Thailand

**Keywords:** employee incentives, job performance, service quality, patient satisfaction, business cycle, population health, health performance, economies

## Abstract

In the past, different researchers have conducted studies on incentives and how they are linked to employee motivation, influencing emerging economies. This study addresses two gaps as outlined in previous studies. One research gap exists in examining employee loyalty and employee engagement in relation to the business cycle. The other gap is observed in the recommendation that future researchers use different moderators between incentives, the health of employees, and job performance with population health. This focus was explored in the present study by identifying the responses of hospitals and physicians to the business cycle to examine the impact of incentives on job performance and health of workers in public and private sector hospitals in Shandong, Eastern China. Data were collected in the form of questionnaires that consisted of close-ended questions. These questionnaires were then filled out by 171 doctors and 149 nurses working in both public and private sectors in Shandong, Eastern China. The results showed that there is a relation between different variables. Some variables have more impact on other variables such as transformational leadership, which has a significant impact on the job performance and business cycle, whereas monetary incentives also impact job performance and population health, but this impact was lower than that of transformational leadership in terms of how job performance influences emerging economies.

## Introduction

The population of China is continuously increasing day by day. In 2019 it was 1.4 billion and is growing at a rate of 0.43% (1). With this population growth, there is a need to focus on the health facilities that are being provided to people and the employees and workers of the health sector. Public sector hospitals are trying their best to provide the best facilities to citizens and their employees but they are lacking in resources compared to the private sector, particularly in terms of technology and staff (2–4).

Through this comparison of both public and private sector hospitals, we can also see the reasons why researchers believe that the public sector has low productivity. These include unfriendly and unprofessional care and an absence of performance based incentives (5, 6). This might be linked to how hospital staff feel about their own health and organization. The present study explores employees' general health and the loyalty of employees regarding the hospital sector (7). This will help the government sector to adopt some techniques that are being used in the private sector and create a working environment that is more conducive to the health of employees, enabling them to be more productive.

Organizations are trying to adapt according to the changes in the environment such as those caused by the Covid-19 pandemic (8). For that purpose, they are focusing on a resource-based view. They are trying to get a competitive advantage by creating human capital that is valuable, rare, and difficult to replace. In doing so, they are making sure that the employees' needs, i.e. health, a healthy environment, and incentives are being fulfilled (9). Organizations believe that fulfilling these needs will lead to employee loyalty and later on affect their general health and job performance. Leaders also play a role in creating an environment that promotes the good health of employees and loyalty of employees leading toward job performance (10, 11). Therefore, this study focuses on exploring whether incentives are the reason for the increase or decrease in an employee's general health and loyalty. It also tries to evaluate the impact that incentives have on job performance and the health of workers. This study also explored the role of leaders in creating employee loyalty and in job performance (1, 12). This study also explores the impact that leaders have on incentives, and how personal care can affect job performance.

Employees are assets of an organization and influence sustainability in organizations. Organizations use various strategies to retain employees and for that purpose, they have different policies in the organization that recognize the efforts of those employees (13, 14). They provide them with rewards or incentives so they may live a healthy life that enables them to contribure to the organization in positive ways. These incentives are there in order to make employees motivated and increase productivity. The rewards system is one beneficial policy for employees, encouraging them to improve and maintain their general health and job performance (15).

Different researchers have examined motivational incentive and reward systems for employees. Some researchers believe that these incentives are the reason employees feel energetic and motivated toward their work. However, some researchers have also focused on the effect that a positive environment has on employees. If organizations provide them with this environment, then employees help them in achieving their goals. When an organization is developing, they try to motivate employees by training or providing them with different facilities including healthcare (14, 16–19). Thus they utilize the skills of employees in different ways. A number of past studies have focused either on the incentive perspective or the environment and how these are linked to determining job performance. Environmental factors are important when it comes to job satisfaction as most employees are satisfied if they have more developmental opportunities rather than extrinsic rewards (20–22). If they get the best out of the prevailing conditions, for example staying healthy during Covid-19, they will certainly do their best (3, 4, 23).

In the changing environment, organizations are trying to adapt. They need to understand the importance of both working environments and extrinsic rewards (24, 25). If one of them is missing, then employees will not be satisfied with their work and that dissatisfaction will lead to bad health and an increase in turnover. Therefore, this research focuses on both perspectives side-by-side, exploring incentives and the environment in relation to leadership as these determine general health, employee loyalty, and the job performance of employees within an organization (12, 21, 26).

This research was conducted in public and private hospitals in Shandong Province, Eastern China. This research will help in determining employees' perspectives regarding incentives and leadership and how they affect their health and loyalty toward the hospital as well as their performance. This research will help different HR managers working in the hospital sector and in other sectors to understand that it is important to focus on both these perspectives and what things are needed to focus more on job performance, better health, and how these are linked to organizational failure or success (27–29). This research furthers understanding of employee perception and weightage and how they influence incentives and leadership in the workplace.

This research is based on knowledge gaps identified in two previously published articles. The first article examined “The effects of organizational culture and leadership style on employee engagement and what their impact on employee loyalty is.” In that article, the researcher recommended that future researchers further explore different perspectives of employee loyalty and employee engagement, whether it is in an organizational or an individual context. They also outline that different factors need to be encouraged that lead to employee engagement, such as the leadership styles, as employee engagement has a positive correlation with employee loyalty. They also mentioned that future researchers can use different moderating variables in research. The second article was on “Employee participation, performance metrics, and job performance: A survey study based on self-determination theory” (30–32) published in 2017. This article recommended that future researchers concentrate on the different moderators that may explain the different relationships between incentives, general health, and performance (29, 33–35). They also recommend that future researchers provide an overview of different tasks or their environment as moderators.

## Literature Review

### Concepts and Definitions

#### Incentives

Incentives are defined as concrete incentives or any kind of compensation that is given to an employee in the form of cash. Incentives can also be defined as the objective criteria where an individual simply wants to establish quantifiable standards for performance. Some researchers have divided incentives into two types, namely concrete and moral incentives. Moral incentives refer to indirect compensation through certification, for example appraising someone (36). Concrete incentives refer to a direct way of compensating one's effort by giving a bonus. In this research, we will be talking about the concrete incentives that are being given to workers. According to the American Compensation Association, compensation is defined as the cash and non-cash remuneration provided by an employer in exchange for the services that are provided by the employee (37). This research discusses concrete or cash remuneration.

A compensation package is when an incentive is used as a strategic tool to compensate an employee for their performance and retain them by achieving employee satisfaction and improving their health for achieving the best job performance at the same time. Some researchers believe that incentives are used by employers to trigger and influence the motivation of employees (38). When they motivate employees, it leads to improvements in general health, skill and they will also be satisfied with their work. Most organizations perceive incentives as a way of achieving their goals (30, 36, 39). Some researchers have outlined that compensation was an important factor in providing health allowances, job satisfaction, and employee empowerment are also considered to be important factors in cases of employee loyalty (28, 30, 39–41).

Some previous research found that the alignment of the reward system must follow the organization's design, otherwise, they send mixed messages (42). Let us take an example of an automobile company, which designs distinct working activities as project assignments for the workforce in departments. The main objectives are that employee pay must be analyzed by the respective managers of departments and decided with a finance manager. Consequently, workers will be satisfied and motivated to meet their functional goals. Although, all the departments must communicate and work cross-functionally. Everyone is responsible and accountable for the responsibilities for which they were hired. The direct supervisor is linked with the incentives that are being provided to the employees rather than a person who has no knowledge of the work performed by the employee. In this way, the employee is motivated and puts effort into working efficiently to gain rewards (43). Therefore, choosing a leader who will measure the performance of the employees is also important.

The reward in satisfying the needs of employees, for example, the healthcare of their family, as stated by Victor H. Vroom under the theory of expectancy (44). Incentives were considered as a form of payment that is directly linked to the performance of employees. The more profits or incentives the better the performance of employees. This system of providing monetary incentives to employees is another way of compensating them other than their salaries. This system of compensating employees is based on performance (35, 37, 45, 46). Different research also shows that employees nowadays are much more motivated by extrinsic rewards. If they have a greater sense of entitlement, then they want to work hard to achieve goals. Employees are not motivated by intrinsic rewards and are more sensitive about monetary compensation for the work that they do (39, 40, 44, 47).

#### Employee Loyalty

Employee loyalty is defined as the commitment or psychological attachment of employees toward the organization. Employee loyalty is also defined as the capability of the employee to stay in an organization. It might also depend on how much time they have spent in an organization and what type of work they do in an organization (46, 48, 49).

In the past, employee loyalty was defined as the time an employee remains in an organization but due to the environmental changes during the pandemic, its definition was updated to the time an employee remains committed toward the organization and is said to be loyal. Previously, employee loyalty consisted of two major divisions: firstly, loyalty is in the employer's best interest; and secondly, loyalty is when an employee remains with the same employer (50). Employee loyalty has evolved. Traditionally, it was known as a trust and bond relationship between employee and employer, which means the longer the time an employee spends on their job the more loyal they will be but in recent times leaders do not equate longevity with loyalty, rather they define loyalty as the commitment and dedication that an employee gives it to their organization (51, 52). Employee loyalty is considered to be linked with the survival and success of any organization and if the employers recognize the importance of an individual, then it means that they will try to ensure that the employee remains loyal to that organization (53–55).

Some researchers believe that employee loyalty leads to job satisfaction. If the employee's expectations are met, then their level of satisfaction also increases. This loyalty will develop into a generalized emotional attitude toward that organization for which the employee is working (56–59). The more satisfied they are the more healthy their life, and the more loyal they will be toward that organization. When employees develop affection toward their organization, they show loyal behavior by improving productivity and that helps them in achieving organizational goals i.e. they provide a better quality of services to employees whereas, some researchers have mentioned that employee loyalty is generated due to the presence of job satisfaction (57, 58, 60, 61). Job satisfaction and the general health of employees are important variables when it comes to employee loyalty. The positive or negative feelings of employees might later determine employee loyalty and eventually affect their performance. When employees have satisfaction they become committed to that organization (8, 23–25, 62) and will remain loyal. A study showed that if the banking sector continued to provide proper compensation, training, and appraisals then the employees will remain committed to the organization and the chances of turnover will also be less. In the past, different studies have found out that different employee loyalty behaviors were linked with how much employees were satisfied with their job, which lead to a commitment to the job (63). Job security was also one of the reasons employee loyalty is generated in some individuals (64).

Past research has outlined that employee loyalty has nothing to do with the human resource management policies and practices, environmental conditions such as a healthy atmosphere. How much an employee is satisfied with their job will later determine the employee loyalty that they develop (65–67). Employee satisfaction is quite important when an employee is from services or sales departments as if these employees are not satisfied with their job then customers are not satisfied with the services they are providing, which will affect the company's goals and impact loyalty toward the organization (64). In this case, employee satisfaction leads to them being loyal toward the organization. If they are not healthy and satisfied with their work not only do they fail to develop employee loyalty, it will harm the organization's performance as well (68, 69). Thus, job satisfaction influences employee loyalty, and later on impacts organizational commitment as well.

Leadership can be defined by the behaviors employees possess and how they process their decision-making. A leader is one or more people who either select, train, or influence their employees. They have a diverse set of abilities, skills, and knowledge that helps them to align employee goals with organizational goals. A leader is a person who influences the behaviors of employees or their followers (70, 71). A leader is also defined as a person who has the ability to understand and work within a culture, which makes a leader effective. Leadership style is defined by the psychological latitude and the behavior they possess during interaction with employees or while they are handling their operations or activities (72, 73). In this research, we examine transformational leaders in order to explain the leadership style used in organizations. Transformational leaders are defined as those who motivate their followers to achieve goals through inspirational motivation, intellectual stimulation, idealized influence, and individual consideration (70, 71, 74). They help the followers perform better than expected. They possess good visioning and use their skills to develop a strong bond with followers. Transformational leadership is used to describe the situation where leaders and followers help each other reach higher levels of morality and motivation (75–79).

Past research has focused on the leadership personality traits that made people successful leaders but some researchers believe that leaders have some innate qualities that distinguish them from other people. Research has started to focus on the different behavioral aspects of leader's personality and the different contingency theories that support situational leadership outline that leader effectiveness depends upon situational factors (75). Other researchers have defined leaders based on two types, including effective leaders and transformational leaders. Transformational leadership is when leaders motivate their employees to increase or strengthen their perceptions, behaviors, commitment, motivation, and beliefs to stay aligned with organizational goals. Whereas, an effective leader is someone who influences followers in such a way that leads to an organizational vision that sets an example by performing a job in such a way that inspires the followers. In other words, an effective leader is someone who leads through their actions (11, 20, 80).

Different leadership styles are seen to be highly supportive and engage employees in their decision making. This engagement in different activities of work makes employees happy that their decision and participation are valued in the organization, which makes them more loyal (81). Different research has shown that employee engagement leads to organizational success (7). Therefore, leader communication strategies have a significant impact on employee general health and their loyalty or commitment ultimately affects job performance in an organization (82). Today, worker loyalty is one of the utmost factors in the success of an organization. For this reason, key leader communication strategies are taken into account in the present study to determine how a worker's motivation loyalty can be increased through different leadership communication strategies, which results in an increase in job and organizational performance (13).

#### Job Performance

Job performance is defined as a certain behavior that organizations expect an individual to carry out. Different researchers have defined job performance as a multidimensional concept that includes both task performance as well as contextual performance (81). Task performance is defined as an employee's contribution toward the organization i.e., their technical competencies and job proficiency, whereas contextual performance is not linked with the formal job requirements of an employee (32). This article focuses on the task performance of the employee. Here, performance includes the outcomes of a particular job that an employee is performing at their workplace. Thus, it is more linked with the task performance of an employee. It is also linked with the employees' behavior toward their work.

Performance is composed of many other different concepts but on a basic level. It can be described as behavioral engagement with an expected outcome, where behavior shows the action people perform to complete the work, outcomes exhibit the results of individual job behavior. Performance is considered a multi-dimensional concept. Job performance has received research attention in the last few decades (41). Effectiveness of job tasks involves evaluating the results of employee performance (i.e., financial value of sales). In comparison, productivity is defined as the ratio of effectiveness to the cost of attaining the outcome. For example, the ratio of hours of work that an employee is investing as input and the product they assemble as output both describe the productivity of an employee. Therefore, performance must be evaluated separately from efficiency and effectiveness in productivity (29).

##### Leadership as a Moderator Between Employee General Health, Loyalty, and Performance

Different leadership styles and strategies are used by organizations to improve employee loyalty and the overall performance of the organization. Different past studies have examined the impact of leadership and these impacts vary according to their styles and the effect the employee has on commitment or loyalty, which significantly affect job performance (40). Different researchers have also explored how leadership style has an impact on organizational culture and organizational performance. Different studies have shown that in the past, leaders inspire followers to accomplish certain organizational goals but in recent years it has been observed that leaders have failed to motivate employees as employees are much more focused on the concept of working to live and are thus more focused on rewards (45). This is due to the fact that employees are so involved in their work that they forget to take care of their health. The researcher has also talked about different research that has claimed that employees growing value is much more on extrinsic rewards and they are not motivated by the charisma of any kind of leader as they want to seek outcome. About 70 % of employees thought that they would get promoted within 2 years in a firm (83). This also shows that employees have high expectations from the firm where they work. Other researchers have found that servant leadership style has a positive relationship between employee loyalty and servant leadership style. Different leadership styles might have a different relationship with job performance depending upon the situation or the context that employees and supervisors are in. This also shows that without a leadership style the link between employee loyalty and job performance can decrease (37, 38). Leaders can create an environment for them to be motivated toward the achievement of goals. If leaders do not guide employees, they might be de-motivated and think that they are not getting incentives, but a leader tries to make sure that employees understand their work and that their contribution will pay off in the form of incentives.

##### Incentives and Employee Loyalty

Different researchers have examined the loyalty of employees. Some of them have used hotel managers and supervisors to check loyalty. They found out that loyalty was associated with intangible aspects. This intangible aspect can be the working environment of the organization and this environment was linked with their peers, supervisors as well as customers. They were satisfied with their jobs because they had opportunities for personal healthcare, development, and to use strengths that helped them in achieving their work objectives (31). They also said that employee turnover was related to there being no opportunities for development. This study focused on how employee loyalty was linked to environmental factors that were creating motivation among employees, leading to them achieving their goals. In the past, it was believed that performance and employee loyalty was linked with promotions. But later on, the focus shifted toward the relationship between the employee and employer and became much more focused on flexible environments to get better performance from employees, as they felt more energetic and healthy when working. Employee loyalty reduces the turnover intentions among employees. The hospital industry has suffered an increasing rise in employee turnover, with the main reason being poor wages (45). Another study showed that employee loyalty was used as a mediator between commitment and employee retention and the results of that study showed that it had a significant effect on both commitment and employee retention. The study mentioned that compensation and different social benefits have a greater impact on employee commitment rather than on retention (37). One of the most important factors should be taking care of the health of employees in changing environments such as during the Covid-19 pandemic. Most of the time, organizations focus on giving financial rewards to their employees but they sometimes forget that non-financial rewards are also important to keep employees motivated toward work. Therefore, both incentives are important when trying to motivate employees and we need leaders to create an environment for the employees that enables them to feel motivated toward work (84).

Some researchers have tried to explain incentives and employee loyalty from a different perspective. They have talked about how employment and unemployment rates can change an organization's point of view (85). One study mentioned that when there is a higher rate of unemployed in the country then the chances of the potential job loss become more, at that time the employer gives employees incentives to gain loyalty. Organizations try to avoid layoffs and at that time they need more loyal employees and for that purpose, they provide stronger incentives. This shows that incentives depend on the leader or the organizations and much they are encouraging employees to perform better. This can also depend on the availability of human capital and when human capital is not easily available in the market, meaning the employer tries to gain employee loyalty by giving out benefits to retain talent in the organization (76).

##### Employee Loyalty and Job Performance

Employees' work attitudes can predict their outcomes. The main dimensions linked to employee loyalty are incentives, healthcare facilities, salaries, promotions, and different individual characteristics such as the age of the employee, job tenure, and position. Different studies have focused on perceived organizational support, customer participation and perceived that supervisory support can lead to an increase in job satisfaction and later on, also improve the service quality of employees (34). Employee loyalty is determined through leadership, human relations, personal development, better health, creativity, and job satisfaction. The better these determinants are handled the more employee loyalty indirectly affects job performance in other ways, as there is a positive relationship between employee loyalty and job performance (38).

##### Employees Incentives and Job Performance

It is suggested in literature on human resource management and organizational behavior that nonmonetary incentives act as a tool for motivating employees. When organizations pay attention to different monetary tools i.e., paid leave, giving bonuses for having an eye on their health and their family healthcare or other family, then employees start to perceive that the organization is supporting them. Therefore, monetary incentives increase the motivation of employees leading to increased job performance (78). Non-monetary tools can be appraised by the leader or environment that leaders provide to their employees. These non-monetary tools keep employees motivated for a certain time but if organizations do not give proper incentives to employees then it will affect their work. Different studies also show that incentives play a part in the job performance of employees. A study investigated the link between incentive packages and employees' attitudes concluded that several different types of incentives (monetary, tangible, and non-tangible non-monetary) play important roles in enhancing employees' attitudes toward their work. Different studies have found that there was a linear correlation between employee loyalty and job performance (72).

Another researcher discusses how job satisfaction impacts employee loyalty (76–78). They state that different underlying factors affect job satisfaction, including healthy working environment, healthy activities, chances of career growth, and motivation, all of which lead to employees being loyal. Some researchers believe that job analysis, compensation, and career planning help to determine employee loyalty. These factors motivate employees in the workplace and further lead to an increase in job performance. However, an absence of employee loyalty can create different issues like an increase in turnover rate among employees (71). Organizations might not be concerned about losing bad performers but if they lose good performers then that is a major concern for the organization. Therefore, the organizations are more focused on retaining human talent in the organization by giving out different facilities to employees and their families. Later on, the benefits incurred by employee loyalty will be more from the cost that was invested in them. Various studies have also shown that employee loyalty is linked to customer services and developing customer loyalty to ensure long term profitability for the organization (73).

Expectancy theory by Victor H. Vroom suggests that people put effort into work when they start to perceive that it will lead to an increase in their performance, which will eventually increase the chances of them receiving rewards. Consequently, an increase in these financial incentives also enhances employee loyalty, which increases the employee's performance and reduces the turnover rate (86). Employees can only be loyal when their desires are being satisfied by organizations. The organization also pays attention to these things, as they also believe humans are an asset and that they need to fulfill their needs to utilize their skills. In the hierarchy of needs (1954), Maslow concluded that humans have five basic wants (physiological, safety needs, love, and belonging, self-esteem, and self-actualization), which can be satisfied through financial incentives and rewards. Employees with a sense of recognition from their employers fall under the heading of self-esteem and, as their needs are being fulfilled, they will experience increased job satisfaction as well (87–89).

##### Theoretical Reflection

Two micro theories support the conceptual framework of our study. These motivational theories explain why incentives influence employee loyalty, leading toward employee performance. The first motivational theory is “Maslow's hierarchy of need,” which classifies human needs into two types. First, lower order needs, which are physiological and connected to safety and security, and second, higher order needs, which include socialization, self-esteem, and self-actualization. In this theory, incentives and this type of recognition are given to employees, and are related to the self-esteem of employees. These create motivation among them to work hard within the organization. The second motivational theory is by Herzberg, who explains two types of factors i.e., motivational factors and hygiene factors (90). Employees would like to grow in an organization and if there are chances for growth, advancements, and recognition they feel motivated to work but hygiene factors like working environment, quality of interpersonal relation, and salary are also important along with the motivational factor (91). Without any one of them, an employee will start to feel dissatisfied with their job, which will impact their behavior toward work and can also lead to high turnover among employees (92).

**Figure 1:**
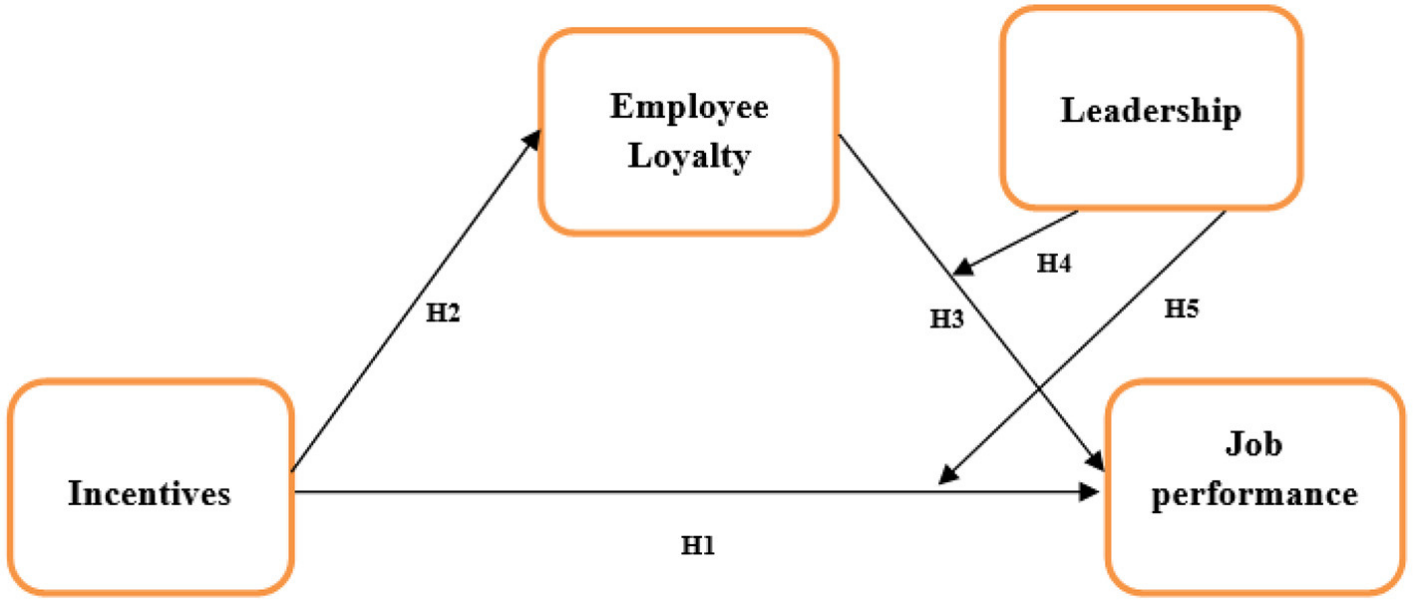
Conceptual framework.

Another important theory that is highly relevant to our study is “Vroom expectancy theory.” This theory suggests that behavior will develop certain attitudes among employees, which will lead to further actions. This theory outlines that job performance is based on certain things i.e., skill, personality, experience, abilities, and knowledge regarding that particular field (93). The effort an employee puts into work; performance and motivation are all linked to employee motivation. This model uses three variables, including expectancy, valence, and instrumentality. Expectancy is defined as the belief about how much effort an employee puts into their work that will lead toward increased performance (90). Valance is defined as the importance an employee gives or places on the expected outcome. Instrumentality is defined as an employee's belief that if they do well in an organization, a valued outcome will be received. In this study, an organization motivates employees to work well and when they do so they receive incentives for the work they have done. Employees feel motivated by these rewards, which creates employee loyalty among them as they think organizations care about their contribution and their needs at the same time. If organizations do not provide incentives to employees who have performed well in the organization, they will feel demotivated and their performance will also decrease, and they will not trust the organization's rules and procedures, potentially decreasing employee loyalty as well (94). Organizations try to motivate their employees by either providing them with a good healthy working environment and in this case, with a leader who will make sure that the working environment motivates an employee to increase loyalty and job performance. Therefore, there is a continuous cycle connecting staying healthy, job performance, incentives, and rewards to future job performance. The incentives or rewards determine whether the employees are motivated enough or, if they are not motivated by the rewards given or think that the incentives given to them do not reflect what they have contributed to the organization, then their performance will decrease in the future (95). Based on the discussed literature, we formulated a conceptual framework, shown in Figure 1.

##### Conceptual Framework

**H1:** Incentives have a positive effect on employee loyalty.

**H2**: Employee loyalty has a positive effect on job performance.

**H3:** Leadership can have a positive moderating effect on employee loyalty leading toward job performance.

**H4:** Leadership has a moderating effect on incentives and job performance.

**H5**: Incentives have a positive effect on job performance.

## Research Methodology

This research focuses on a quantitative methodology. Quantitative methods are focused on a systematic way of collecting data either through questionnaires or surveys. Quantitative research explains the phenomenon according to numerical data. It is also defined as the empirical research that explains a social phenomenon by testing a theory consisting of different variables. Researchers want to explain the perception of employees regarding incentives, healthcare, employee loyalty, leadership, and job performance in the language of statistics and mathematics. In this research, the researchers have only focused on the hospital staff's point of view regarding this matter and have collected data from them for this research. This research was a quantitative, descriptive, and cross-sectional study. The overall methodology of this study is positivism, as it describes the study through different statistics that are gathered by collecting data.

### Research Design

**Figure 2:**
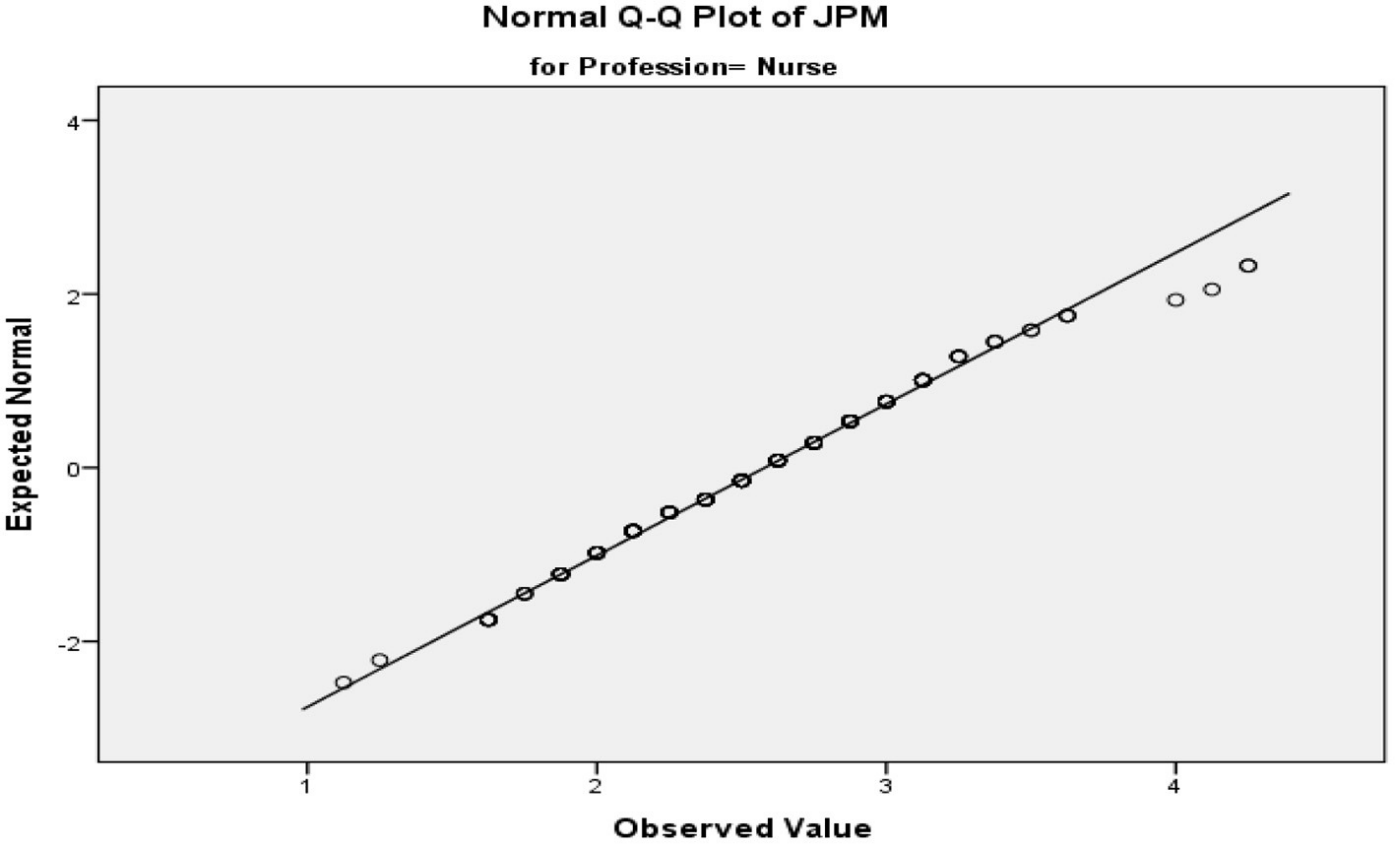
Normality plot of JPM.

The research design provides an outline for research. It also provides a guideline for the researchers who are performing that research (96). While this research focuses on how incentives can lead to employee loyalty and further contribute to job performance, this research also focuses on the impact leaders have on employee loyalty and job performance. Thus, it tries to describe the relationship between different variables, how they impact each other, and which variable has the most impact on the other variables. Therefore, this research used quantitative methods of collecting data by giving out questionnaires to the hospital staff. Quantitative research was used for this research to make the data more representative and to generalize the information collected and examine the hypotheses proposed in the literature section. These empirical results could also help future studies of other developing countries with similar work conditions. Figure 2 shows the Levene's test of equality of error variances.

### Research Approach

This research used a deductive research approach, which converts specific things into general applications. In the deductive approach, the researcher first finds a theory related to the conceptual framework and then analyzes that data (10). This will help in interpreting the data but also in explaining data presented in the form of graphs and numbers. This deductive approach will help us explain the results through different theories. It will also help in accepting and rejecting the hypotheses and explain why people thought different variables were affecting each other.

### Data Collection

The data for this research were collected from primary and secondary sources. Primary data was collected by giving out questionnaires to hospital staff e.g. doctors and nurses. The questionnaires consisted of close-ended questions. In total, 320 questionnaires were collected from respondents and later analyzed. The reason for using a quantitative method to collect data was to make sure that we gathered enough information from the sample, could easily compile data regarding employee perceptions, and easily analyze the data gathered. Where the secondary data was gathered through several sources i.e., articles, and books, the secondary data were used to formulate the literature review and support the description of findings.

### Population and Sampling

A population is defined as a group of individuals that have certain skills, knowledge, or experiences required for research. The population chosen for the present research included staff members working in both public and private sector Hospitals in Shandong, eastern China. The term population is also defined as a wide range of people. Every study is based on a certain population e.g., hospital sector, banking sector, or schools, but collecting data from such a huge number of people is impossible. For that reason, researchers divide this population into a sample to easily collect the required data. Certain techniques are used by researchers to select samples.

The sampling technique used for the collection of data was cluster random sampling. Clustering is a useful method of collecting data and discovering different groups of respondents that represent the population. The sample might be taken from a particular city or a particular sector. These clusters help in dividing the greater population into smaller sections. The later helps in separating people with similar patterns. Clustering does not indicate the desired relations that would be valid among the data beforehand and for this reason, it is thought to be an unsupervised process (24).

The sample taken for this study is forms of hospital staff i.e. doctors and nurses from both public and private sector hospitals of Shandong, Eastern China.

Data were collected via questionnaire to see what impact incentives have on their job performance or their employee loyalty and what type of leadership was being provided to them. The questionnaire was based on the Likert type scale.

### Data Analysis Techniques

For the analysis, data were first screened to see if there were any missing values. Data collected through questionnaires were coded and then analyzed using SPSS. The data gathered from participants were gathered through Likert type scale questions and was dissolved into high and low groupings and positioned on scales 1 = strongly agree and 5 = strongly disagree. The respondents that rated 1–3 were considered high, whereas ratings from 4 to 5 were considered low. The data were analyzed in SPSS by applying different tests such as correlation, descriptive analysis, normality tests, and ANOVA to analyze the gathered data. Furthermore, confirmatory factor analysis (CFA) was also performed to analyze different variables.

## Data Analysis

### Descriptive Analysis

Descriptive analysis justifies the salient features of the study and provides a comprehensive summary of the data used in the study and also shows different statistical measures. Collectively, with simple graphical analysis, they form the structure of quantitative analysis of data. Descriptive statistics are easy to understand for general readers and show the behavior of the data. It is also used to present the data in a manageable form. Descriptive statistics cover the different aspects of the data i.e., central tendency, the measure of dispersion, the measure of normality, and trends in the data. The section covers the results descriptive statistics for the hospital sector in Shandong, eastern China. In Table 1 data are analyzed using SPSS software, which illustrates the total number of observations, arithmetic mean, standard deviation, the maximum and minimum value of each variable, which provides an entire description of the data used in the study.

**Table 1:** Reliability analysis.

	**N**	**Range**	**Min**.	**Max**.	**Mean**		**SD**	**Variance**	**Skewness**		**Kurtosis**	
	**Statistic**	**Statistic**	**Statistic**	**Statistic**	**Statistic**	**SE**	**Statistic**	**Statistic**	**Statistic**	**SE**	**Statistic**	**SE**
ELM	320	2.67	1.00	3.67	2.15	0.030	0.537	0.289	0.077	0.136	−0.098	0.272
TSLM	320	4.00	1.00	5.00	2.52	0.047	0.853	0.729	0.402	0.136	−0.228	0.272
MIM	320	4.00	1.00	5.00	2.82	0.040	0.717	0.515	0.200	0.136	0.061	0.272
JPM	320	3.13	1.13	4.25	2.49	0.033	0.591	0.350	0.238	0.136	0.402	0.272
Valid N (listwise)	320											

*Min, Minimum; Max, Maximum; SE, Standard error; SD, Standard deviation; N, Total sample*.

Table 1 represents descriptive statistics for the hospitals in Shandong, eastern China for a total of 320 observations. The mean value of the job performance is 2.4948 with a standard deviation of 0.59176. This means that the value of job performance can deviate from the mean to either or both sides of the mean by 0.59176 and the maximum value of job performance is 4.25 with 1.13 as the minimum value. The mean value of monetary incentives is 2.8203 with a standard deviation of 0.717, which again means that the mean value of monetary incentives can deviate to both sides of the average by 0.717 with a maximum and minimum value of 5 and 1. Furthermore, the table shows that the mean value of employee loyalty is 2.1538 with a standard deviation of 0.537 with a minimum and maximum value of 3.67 and 1 in contrast, transformational leadership has a mean value of 2.6167, with a standard deviation of 0.853 and has a minimum value of 1 and maximum value of 5.

The correlation table above clearly shows that the variables used in the study are related to one another, in other words, the independent variable influences the dependent variable. Simultaneously, the mediating and moderating variables also influence the independent and dependent variables. The tables above illustrate that there is a 0.180 correlation between job performance and monetary incentives, which means that if monetary incentives are increased by 1% job performance will increase by 0.180% hence it has an impact on job performance. Furthermore, the correlation matrix states that transformational leadership does influence job performance and has a greater correlation with dependent variables with a value of 0.222, apart from this, employee loyalty also has a positive correlation with job performance, with a value of 0.240 apart from this, the value of correlation is significant because the *p* value is < 0.05. Hence, statistics prove that the variables do have a relationship.

### Descriptive Analysis

Normality is one of the assumptions of running the ANCOVA model on data. It states the error between observed and predicted values are normally distributed. The hypotheses for the normality are as follows (see Table 1):

Ho = the error term is not normally distributed.
H1 = the error term is normally distributed.

To use the 320 observations from the hospital sector, data were analyzed to check whether the concerned variables are normally distributed or not.

The normality assumption was checked and is supported by different tests applied to the study, among them Shapiro wilk test is simple, effective, and is a standard test for checking normality (see Tables 2, 3). Observing the size and nature of the data, Shapiro Wilk and Kolmogorov-Smirnov tests were applied to determine the normality of data, as shown in the table below. The table below showed that the majority of the significant values were <5%, which means the data are not normally distributed, but the table also shows that the *p* value in terms of nurses is significant and proves that data are normal (Tables 2, 3). Hence, the null hypothesis has been rejected and an alternate hypothesis is accepted.

**Table 2:** Correlation matrix.

		**MIM**	**ELM**	**TSLM**	**JPM**
MIM	Pearson correlation	1	0.059	0.307^**^	0.180^**^
	Sig. (2-tailed)		0.290	0.000	0.001
	N	320	320	320	320
ELM	Pearson correlation	0.059	1	0.323^**^	0.240^**^
	Sig. (2-tailed)	0.290		0.000	0.000
	N	320	320	320	320
TSLM	Pearson correlation	0.307^**^	0.323^**^	1	0.222^**^
	Sig. (2-tailed)	0.000	0.000		0.000
	N	320	320	320	320
JPM	Pearson correlation	0.180^**^	0.240^**^	0.222^**^	1
	Sig. (2-tailed)	0.001	0.000	0.000	
	N	320	320	320	320

** *Correlation is significant at the 0.01 level (2-tailed)*.

**Table 3:** Tests of normality.

	**Profession**	**Kolmogorov-Smirnov** ^a^			**Shapiro-Wilk**		
		**Statistic**	**Df**	**Sig**.	**Statistic**	**df**	**Sig**.
JPM	Doctor	0.085	171	0.004	0.972	171	0.002
	Nurse	0.062	149	0.200^*^	0.984	149	0.088
ELM	Doctor	0.136	171	0.000	0.975	171	0.003
	Nurse	0.080	149	0.020	0.983	149	0.056
TSLM	Doctor	0.126	171	0.000	0.961	171	0.000
	Nurse	0.131	149	0.000	0.973	149	0.006
MIM	Doctor	0.147	171	0.000	0.962	171	0.000
	Nurse	0.140	149	0.000	0.974	149	0.006

* *This is a lower bound of the true significance*.

a *Lilliefors significance correction*.

### Test of Normality

**Figure 3:**
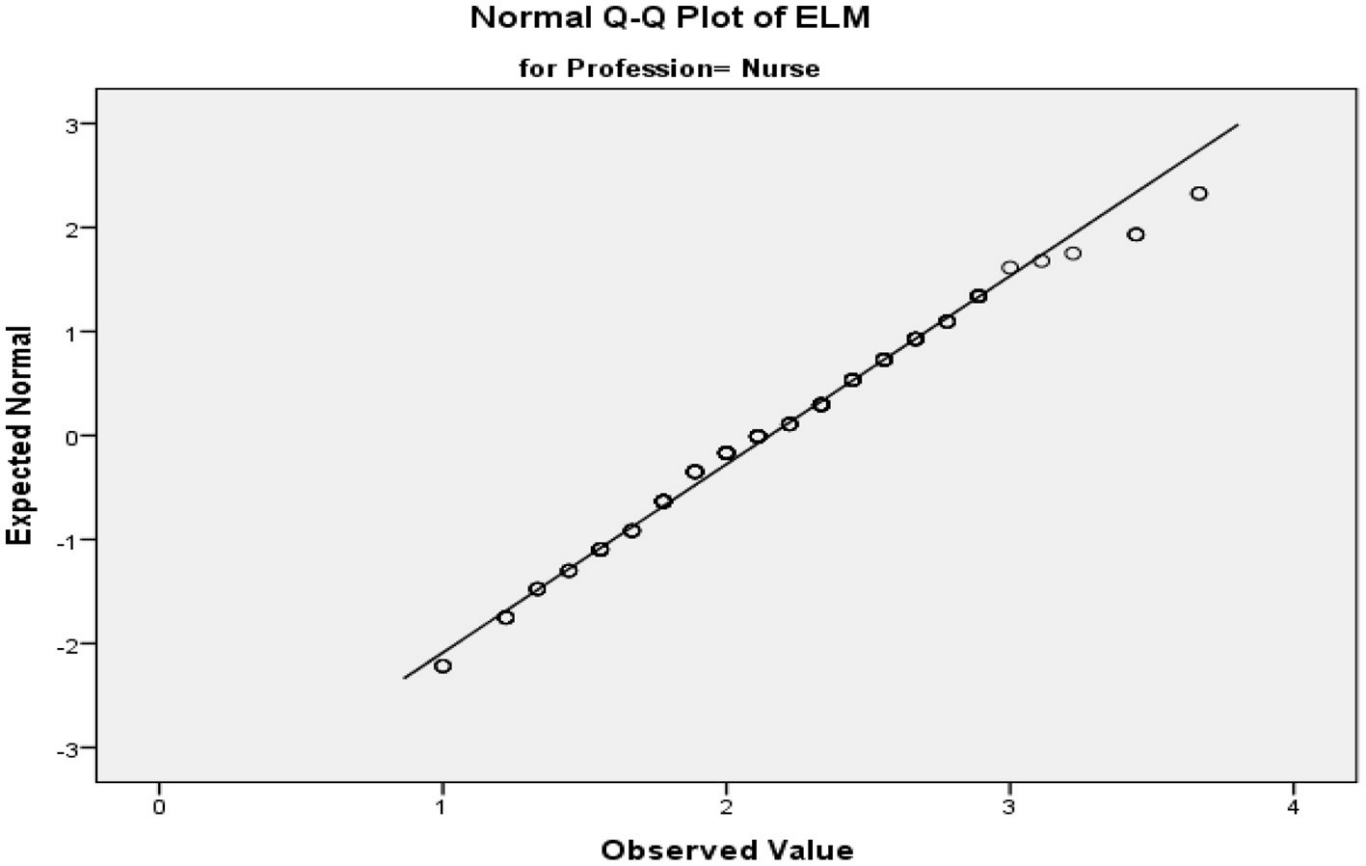
Normality plot of JPM.

**Figure 4:**
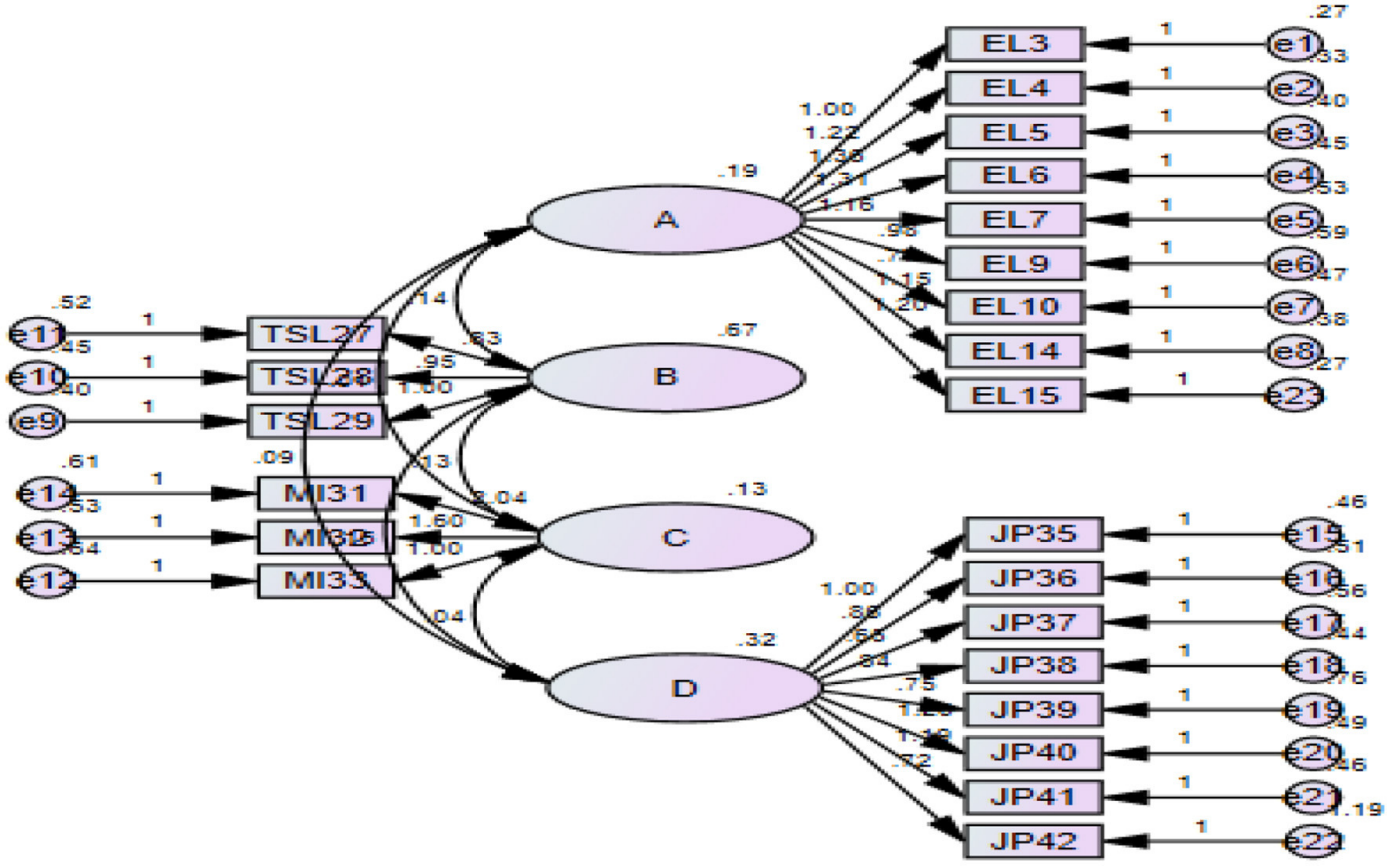
CFA model.

To check the homogeneity of variances of the data collected, Levine's test of equality of error variances was applied (Table 4). The table below clearly shows that the results are significant, indicating that the mean *p* value is < 0.05, which proves that homogeneity of variances exists in the collected data (see Figures 3, 4).

**Table 4:** Levene's test of equality of error variances.

**F**	**df1**	**df2**	**Sig**.
**Dependent variable: JPM** ^a^			
2.779	13	306	0.001

*Tests the null hypothesis that the error variance of the dependent variable is equal across groups*.

a Design: Intercept + MIM + ELM + MIM ^*^ ELM.

### Confirmatory Factor Analysis

The following table reports the analysis between our variables. The coefficient value between depending on and independent variable that is job performance and monetary incentives is 0.04, which shows every unit increase in monetary incentives the job performance increase by 0.04 percent at a 5% significance level. The result shows that employee loyalty plays a mediating role in job performance through monetary incentives at a 5% significance level. Furthermore, the coefficient of transformational leadership between monetary incentives and job performance plays a moderating role of 0.09 at a 5% significance level (Figure 3).

## Results

The data collected in the study were analyzed using SPSS software, regression, and ANOVA analysis techniques because we are measuring the direct impact of monetary incentives on job performance with employee loyalty as a mediator and transformational leadership as a moderator between job performance and employee loyalty. The results are shown in **Table 7**.

### Hypothesis Testing

To check the hypothesis, several values were used, from the model, the basic value used for analyzing the hypothesis are the beta coefficients, Adjusted R square, and R square.

Table 5 clearly shows that healthcare, monetary incentives, employee loyalty, and transformational leadership affect job performance. The value of adjusted R square that is 0.097 means that the 100% fluctuations in job performance out of which 9.7% fluctuations are due to monetary incentives, employee loyalty, and transformational leadership, support the hypotheses of this study.

**Table 5:** Model summary.

**Model**	**R**	**R square**	**Adjusted R square**	**Std. error of the estimate**	**Durbin-Watson**
1	0.311^a^	0.097	0.088	0.56512	1.774

a *Predictors: (Constant), TSLM, MIM, ELM*.

*^b^Dependent variable: JPM*.

Furthermore, the beta coefficients from the model were used to prove each hypothesis separately.

For testing hypotheses 1 and 5, the beta coefficient 0.132 in Table 6 indicates that when the monetary incentives are increased by 1 unit the job performance increases by 0.132. It showed that the value 0.132 is positive, which also proves that there is a positive relationship between monetary incentives and employee loyalty at a significant level of 0.020 < 0.05, hence we accept our hypotheses 1 and 5 (Table 6).

**Table 6:** Coefficients.

**Model**		**Unstandardized coefficients**		**Standardized coefficients**	**t-stat**	**Sig**.	**Collinearity statistics**	
		**B**	**Std. error**	**Beta**			**Tolerance**	**VIF**
**Coefficients** ^a^								
1	(Constant)	1.513	0.177		8.543	0.000		
	MIM	0.109	0.046	0.132	2.346	0.020	0.904	1.106
	ELM	0.213	0.062	0.193	3.419	0.001	0.894	1.119
	TSLM	0.083	0.041	0.119	2.010	0.045	0.812	1.231

a *Dependent variable: JPM*.

Hypothesis 2: there exists a positive relationship between employee loyalty and job performance. The model in Table 6 shows that the beta coefficient between job performance and employee loyalty is 0.193 at a significance level of 0.001 < 0.05, which means that the 1 unit increase in employee loyalty causes the job performance to increase by 0.193 hence our second hypothesis is also accepted (Table 7).

**Table 7:** ANOVA.

**Model**		**Sum of squares**	**df**	**Mean square**	**F**	**Sig**.
**ANOVA** ^a^						
1	Regression	10.791	3	3.597	11.264	0.000^b^
	Residual	100.918	316	0.319		
	Total	111.709	319			

a *Dependent variable: JPM*.

b *Predictors: (Constant), TSLM, MIM, ELM*.

For testing Hypotheses 3 and 4, the beta coefficient 0.119 at a significance level of 0.045 <0.05 in Table 6 shows that transformational leadership does moderate employee loyalty incentives and job performance. The value 0.119 means that when transformational leadership increases by 1 unit positively the job performance of an employee increases by 0.119, which provides logical proof of Hypotheses 3 and 4.

## Discussion and Conclusion

The present study examined the impact of incentives on the healthcare of employees, their loyalty, and job performance. In this study, we used transformational leadership as a moderator to see what impact both incentives and leadership have on the job performance of an employee. The data for this study were collected from doctors and nurses from both the public and private sectors to see the impact that monetary incentives and leadership have on employee loyalty and job performance. The collected data were then analyzed in SPSS through descriptive statistics, correlation matrix, and by doing confirmatory factor analysis and as well as through regression and ANOVA model. After applying these analysis tools several statistics were shown by the model such as the correlation matrix, which proved that a relationship among the variables used in the study exists. All the values in the correlation matrix showed that there was a link between monetary incentives and job performance and that leadership did play a moderating role between incentives and job performance. The correlation matrix also showed that there was a 0.180 % correlation between job performance and incentives including better healthcare. The correlation matrix also showed that transformational leadership had a greater correlation with the dependent variable, apart from the regression model, which gave the healthy justification for accepting all the hypotheses since all the beta coefficients were aligned with the hypotheses and showed a positive relationship among the variables. The key point of the discussion is that this study provides helpful material for managers and employers to understand the behavior of employees regarding their job performance. Organizations could increase employee loyalty by giving meaningful incentives to their employees. Additionally, a good and effective allocation of supervisors to a particular group of employees can increase their job performance and their loyalty toward the organization. Given that the research subjects of this study were health workers from Shandong Province, eastern China, their increased job performance and loyalty to affiliated hospitals could further increase their service quality, enabling higher patient satisfaction.

The first limitation of our study is that it is cross-sectional. The results might be different in the case of a longitudinal study. Therefore, it is recommended that further researchers undertake a longitudinal study on the same variables. Another limitation of our study is that it is quantitative and only describes the relationship between different variables. Future researchers should undertake an in-depth study examining the reasons for the variables affecting each other in this manner. Apart from this, the study was conducted in a few public and private sector hospitals in Shandong and the sample size of the study was small. Thus, future research could use a larger sample size for the same variables. In addition to this, the researchers cannot generalize the findings for this small sample, meaning further research should be conducted in different countries to explore how different factors vary and affect different contexts. Future studies should also compare how these factors affect semi-government hospitals. Further future research could also explore the impact of these variables on administration staff working in the hospital sector.

## Data Availability Statement

The original contributions presented in the study are included in the article/supplementary material, further inquiries can be directed to the corresponding author.

## Author Contributions

WL conceived the main idea and collected the data for analysis. YL suggested the methodology and finalized the manuscript. All authors are agreed on publication.

## Conflict of Interest

The authors declare that the research was conducted in the absence of any commercial or financial relationships that could be construed as a potential conflict of interest.

## Publisher's Note

All claims expressed in this article are solely those of the authors and do not necessarily represent those of their affiliated organizations, or those of the publisher, the editors and the reviewers. Any product that may be evaluated in this article, or claim that may be made by its manufacturer, is not guaranteed or endorsed by the publisher.
